# Alterations of the Host Microbiome Affect Behavioral Responses to Cocaine

**DOI:** 10.1038/srep35455

**Published:** 2016-10-18

**Authors:** Drew D. Kiraly, Deena M. Walker, Erin S. Calipari, Benoit Labonte, Orna Issler, Catherine J. Pena, Efrain A. Ribeiro, Scott J. Russo, Eric J. Nestler

**Affiliations:** 1 Fishberg Department of Neuroscience and Friedman Brain Institute, Icahn School of Medicine at Mount Sinai, New York, NY 10029, USA; 2 Department of Psychiatry, Icahn School of Medicine at Mount Sinai, New York, NY 10029, USA; 3 Seaver Autism Center for Research and Treatment, Icahn School of Medicine at Mount Sinai, New York, NY 10029, USA

## Abstract

Addiction to cocaine and other psychostimulants represents a major public health crisis. The development and persistence of addictive behaviors comes from a complex interaction of genes and environment - the precise mechanisms of which remain elusive. In recent years a surge of evidence has suggested that the gut microbiome can have tremendous impact on behavioral via the microbiota-gut-brain axis. In this study we characterized the influence of the gut microbiota on cocaine-mediated behaviors. Groups of mice were treated with a prolonged course of non-absorbable antibiotics via the drinking water, which resulted in a substantial reduction of gut bacteria. Animals with reduced gut bacteria showed an enhanced sensitivity to cocaine reward and enhanced sensitivity to the locomotor-sensitizing effects of repeated cocaine administration. These behavioral changes were correlated with adaptations in multiple transcripts encoding important synaptic proteins in the brain’s reward circuitry. This study represents the first evidence that alterations in the gut microbiota affect behavioral response to drugs of abuse.

In recent years there has been a growing awareness that crosstalk between intestinal bacteria and the central nervous system, often dubbed the gut-brain axis, is critically important for maintaining brain homeostasis and plasticity^1^,^2^. Several studies have shown that mice born without commensal microorganisms (germ-free), and mice with their gut microbiota disrupted by antibiotics, exhibit behavioral abnormalities in mouse models of several psychiatric illnesses. An early study from Diaz-Heijtz and colleagues demonstrated that germ-free mice have reduced anxiety-like behaviors and altered monoamine turnover in the striatum^3^. In another landmark study, Bercik and colleagues demonstrated that strain-specific anxiety-like behaviors could be transposed simply by transplanting the microbiome between the strains of mice^4^. More recently, several studies demonstrated effects of the gut microbiome in animal models of autism and of depression^5^,^6^,^7^,^8^,^9^. For example, a recent study demonstrated that mice transplanted with gut microbiota from depressed human subjects develop depression-like behavioral abnormalities^5^. While large scale studies of the effect of the gut microbiome on mood and behavior in clinical populations have yet to be published, there is growing evidence that probiotics can alter functional connectivity in the brain^10^ and lead to improvements in mood in some depressed subjects^11^.

Despite this interest in gut-brain connections in mood-, anxiety-, and autism-related disorders, there has been no published work directly examining the link between changes in gut microbiota and the rewarding properties of drugs of abuse. Addiction to cocaine, amphetamine, and other psychostimulants is a major public health crisis that creates a tremendous economic and social burden around the world^12^. These are highly recalcitrant conditions with very high rates of recidivism even after prolonged periods of abstinence. Unfortunately, there remains a dearth of potential therapeutic interventions to help reduce relapse rates. Given the changes in lifestyle and diet that are associated with drug addiction, this is a population that is likely to have altered composition of their gut microbiota which could conceivably be contributing to these maladaptive behaviors. Indeed, a recent study in humans examining the microbiota of cocaine abusers demonstrates significant differences in endogenous microbiota between cocaine users and control subjects^13^. However, it is not yet known if these differences in microbiota are affecting behavioral responses to cocaine.

While most animal studies to date have examined the role of gut microbiota in models of mood and anxiety disorders, it is important to bear in mind that substance use disorders and mood-anxiety disorders are highly comorbid in human populations^14^, and animal models have shown a great deal of similarly affected circuitry and molecular targets in models of both disorders^15^,^16^. Of note, several studies have shown that brain-derived neurotrophic factor (BDNF) is dysregulated in animals with altered gut bacteria^4^,^7^,^17^,^18^,^19^, and BDNF has been shown to play a crucial role in models of both depression and cocaine use disorders^20^,^21^. Additionally, several studies have shown altered levels of monoamine metabolism in the brains of mice with reduced gut bacteria^3^,^18^,^22^ – another change that is of critical importance to behavior related to drugs of abuse.

To test the hypothesis that alterations in the gut microbiota could affect psychostimulant-induced behavior, we substantially reduced the gut bacteria of a group of mice using antibiotics that are not systemically absorbed from the intestines. After this targeted knockdown of gut bacteria, we examined the mice on tests of cocaine preference and locomotor sensitization. We demonstrate that alterations in gut microbiota significantly affect the dose-response relationship for cocaine and lead to alterations in levels of important synaptic transcripts in the brain’s reward circuitry following cocaine treatment.

## Results

### Knockdown of intestinal bacteria using non-absorbable antibiotics

**Figure 1:**
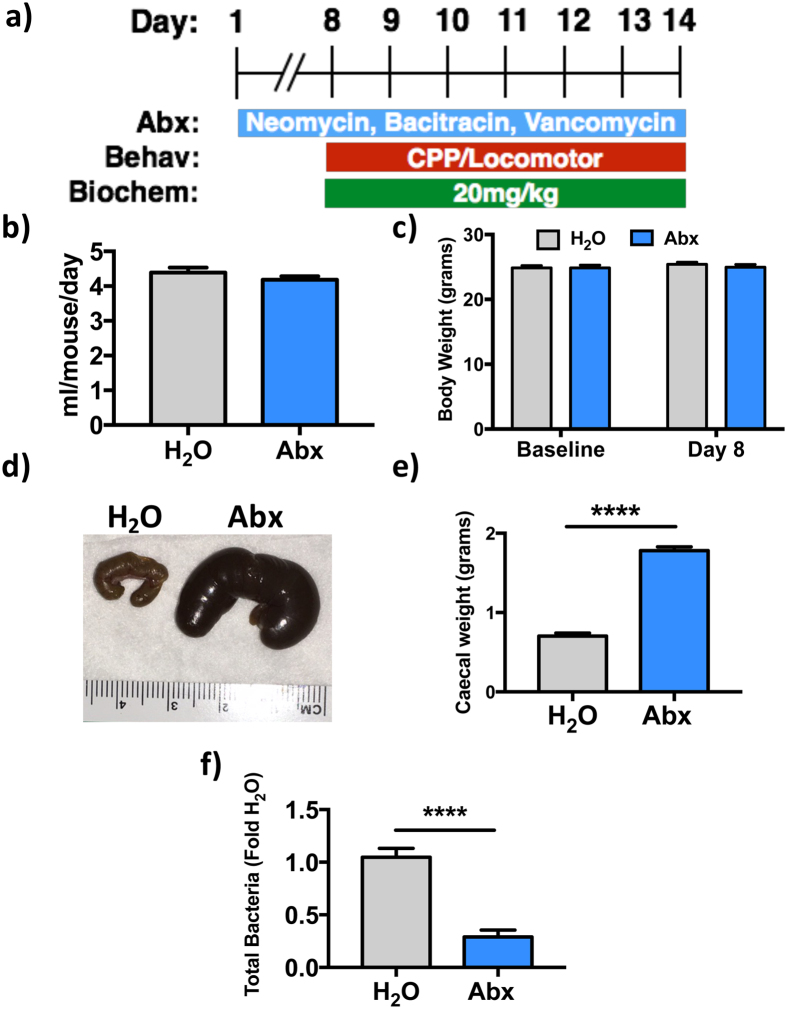
Knockdown of intestinal bacteria using non-absorbable antibiotics. **(a)** Schematic representation of the experimental procedure. **(b)** Fluid intake did not differ between control (H_2_O) and antibiotic-treated (Abx) groups (n = 32 H_2_O, 39 Abx). **(c)** Mean body weight did not differ between groups and was not affected by antibiotic treatment (n = 32 H_2_O, 39 Abx). **(d)** Gross morphology of the cecum from untreated (left) and antibiotic-treated (right) mice with ruler shown for scale. **(e)** Quantitative measurement of cecal weights between control and antibiotic-treated mice demonstrates a marked increase in antibiotic-treated animals (***p < 0.0001; n = 24 H_2_O, 28 Abx). **(f)** qPCR analysis of total bacterial load between the two conditions demonstrates a decrease in bacterial load in antibiotic-treated mice (***p < 0.0001; n = 16 H_2_O, 14 Abx).

To determine if alterations in intestinal microbiota influence the behavioral and molecular responses to cocaine, adult male mice were given a cocktail of antibiotics in their drinking water for 7–10 days before the start of any cocaine treatments (Fig. 1a), a procedure known to markedly reduce gut bacteria^4^,^23^. Daily fluid intake did not differ between groups over the course of the experiment (Fig. 1b **-** two-tailed t-test: *p* = 0.22; t = 1.23), and neither group demonstrated an appreciable change in their body weight during this time (Fig. 1c - two-way ANOVA: *p* ≥ 0.39 for time, treatment and interaction effects). Reduction of the gut microbiota with prolonged treatment with antibiotics resulted in markedly enlarged ceca (Fig. 1d,e – two-tailed t-test: *p* < 0.0001; t = 17.7) as has been previously reported in antibiotic-treated and germ-free mice^23^,^24^. qPCR analysis of 16S rRNA from cecal contents was demonstrated a 72% reduction in bacterial content in mice treated with broad-spectrum antibiotics (Fig. 1f – two-tailed t-test: *p* < 0.0001; t = 7.03).

### Knockdown of intestinal bacteria does not alter cocaine metabolism or serum corticosterone levels

**Figure 2:**
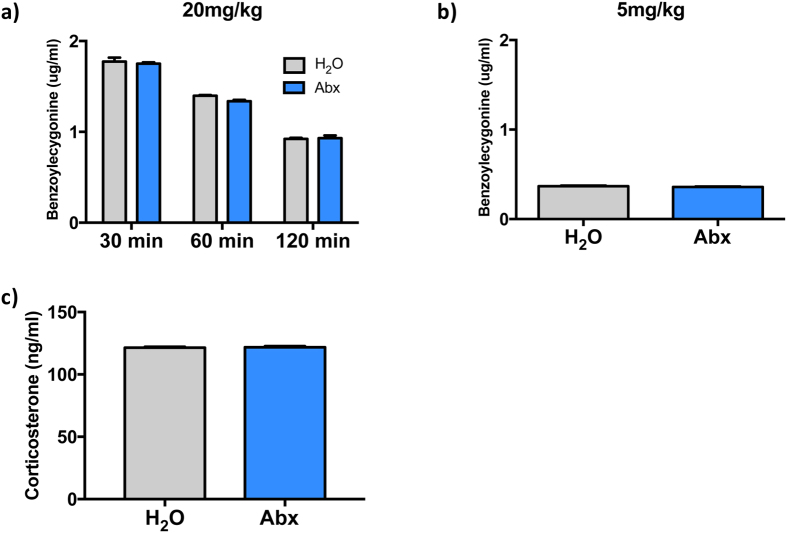
Knockdown of intestinal bacteria does not alter cocaine metabolism. **(a)** Serum levels of the cocaine metabolite benzoylecygonine 30, 60, or 90 minutes after a single high-dose injection of cocaine did not differ between control (H_2_O) and antibiotic-treated (Abx) mice (n = 4–5/group/timepoint). **(b)** Levels of benzoylecygonine 30 minutes after a 5 mg/kg injection of cocaine also did not differ between groups (n = 3 H_2_O, 5 Abx). **(c)** Levels of serum corticosterone in animals treated with antibiotics via the drinking water did not differ between groups (n =  10 H_2_O, 10 Abx).

While the antibiotics used to knockdown intestinal bacteria are non-absorbable and are excreted in the feces, the possibility remained that alterations in gut bacteria could alter the expression of hepatic enzymes which are responsible for metabolism of cocaine – thereby altering the effective dose the animal would receive in a weight-based dosing model. To test this, we measured serum levels of benzoylecygonine, the primary metabolite of cocaine, after cocaine injection. Levels were monitored in a time course after a high dose (20 mg/kg) of cocaine (Fig. 2a). A two-way ANOVA analysis of these results demonstrated the expected effect of time (Time: F_(2,19)_ = 766.3, *p* < 0.0001), but there was no effect of antibiotic treatment (Treatment: F_(1,19)_ = 2.27, *p* = 0.15) or any time x treatment interactions (Interaction: F_(2,19)_ = 1.61, *p* = 0.23). Given that our primary behavioral effects were seen with a dose of 5 mg/kg, metabolism of this dose at 30 minutes post-injection was tested as well (Fig. 2b). As with the higher dose, there was no effect of antibiotic treatment on the metabolism of cocaine to benzoylecygonine (two-tailed t-test: *p* = 0.35; t = 1.01). Taken together, these results show that prolonged oral treatment with non-absorbable antibiotics does not affect the metabolism of multiple doses of cocaine.

We also examined the possibility that our antibiotic treatment regimen may have been stressful and thereby altered function of the hypothalamic-pituitary-adrenal (HPA) stress axis. As serum levels of corticosterone, the primary serum glucocorticoid in mice, can affect behavioral responses to cocaine^25^ we measured serum levels of control and antibiotic-treated mice. From these analyses we see that treatment with antibiotics via the drinking water for two weeks did not alter peak (afternoon) serum levels of corticosterone (Fig. 2c – two-tailed t-test: *p* = 0.68; t = 0.42), which was the time of day when behavioral testing was performed. This indicates that any behavioral changes seen in our antibiotic-treated animals were not likely due to gross dysregulation of the HPA stress axis.

### Alterations in intestinal bacteria affect behavioral responses to cocaine

**Figure 3:**
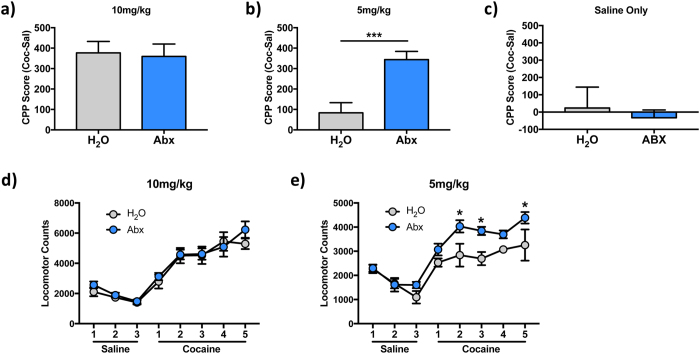
Depletion of intestinal bacteria enhances behavioral responses to cocaine. **(a)** Control animals (H_2_O) and antibiotic-treated animals (Abx) exhibited no difference in place preference for cocaine at a dose of 10 mg/kg (n = 6 H_2_O, 8 Abx). **(b)** Conditioned place preference assay at a 5 mg/kg dose did not produce significant preference in control animals, but Abx-treated animals formed a robust preference for the cocaine-paired side (***p < 0.0001; n = 11 H2O, 22 Abx). **(c)** When both sides of the assay were paired with saline there was no detectable preference or group difference (n = 5/group). **(d)** Both control and antibiotic-treated mice developed locomotor sensitization in response to cocaine treatment, but there were no between group differences (n =  5/group). **(e)** Antibiotic-treated mice show an enhanced locomotor response to repeated doses of 5 mg/kg cocaine (*p < 0.05; n = 5/group).

Changes in gut bacteria have been shown to alter behavior in animal models of anxiety^4^,^26^, depression^5^, and social behavior^7^, but no previous studies have examined how changes in intestinal microbiota might affect behavioral responses to drugs of abuse. To assess how antibiotic depletion of the gut microbiota might affect the rewarding properties of cocaine, we performed an unbiased conditioned place preference (CPP) task for cocaine in mice treated with antibiotics and controls. Initial experiments were performed with a dose known to produce robust CPP (10 mg/kg). While this dose did produce the expected place preference in control animals, there was no difference between control and antibiotic-treated animals at this dose (Fig. 3a; two-tailed t-test: *p* = 0.84, t = 0.21). We next examined a low dose that does not lead to significant place preference in control animals (5 mg/kg). In these experiments we see that the antibiotic-treated mice demonstrate a robust place preference, while the control mice did not form a significant preference (Fig. 3b; *p* < 0.0001, t = 5.71). In control experiments in which both sides of the apparatus were paired with saline neither group formed a preference and there was no effect of antibiotic treatment (Fig. 3c; *p* = 0.67, t = 0.44). These data demonstrate a significant shift in the dose-response curve for the rewarding properties of cocaine in mice with reduced intestinal bacteria.

In addition to the place preference test, we examined the development of locomotor sensitization to repeated doses of cocaine in control and antibiotic-treated animals. Prior to the start of cocaine testing, animals from both groups received three days of injections with saline only to monitor baseline locomotor activity, and to allow animals to habituate both to the locomotor chambers and to the injections. Analysis of these baseline days demonstrated the expected mean effect of time (F_(2,16)_ = 22.95; *p* < 0.001), but, importantly, there were no treatment (F_(1,8)_ = 1.16; *p* = 0.31) or time x treatment interactions (F_(2,16)_ = 1.16; *p* = 0.34) (Fig. 3d - left). This makes the important point that antibiotic treatment on its own does not affect locomotor behavior. When we looked at locomotor activity after cocaine treatment, we obtained results with a similar pattern to those seen with CPP. At 10 mg/kg dosing there was a robust sensitization response across the five-day period for both groups using repeated-measures two-way ANOVA (Main effect of time: F_(4,32)_ = 29.53; *p* < 0.0001). However, there was no effect of antibiotic treatment (F_(1,8)_ = 0.1342; *p* = 0.72) or an antibiotic x time interaction (F_(4,32)_ = 1.486; *p* = 0.23) (Fig. 3d). We then examined a 5 mg/kg dose which generally does not produce a sensitized drug response in this assay. In this experiment, there was a significant difference between the control and antibiotic-treated animals (Fig. 3e - F_(1,7)_ = 19.44; p = 0.003). Only the antibiotic-treated mice showed an increased response over time, with a main effect of time for the experiment (F_(4,28)_ = 3.231; *p* = 0.03). Taken together with the CPP data, these experiments demonstrate that reduction of intestinal microbiota results in increased sensitivity to the behavioral effects of cocaine.

**Figure 4:**
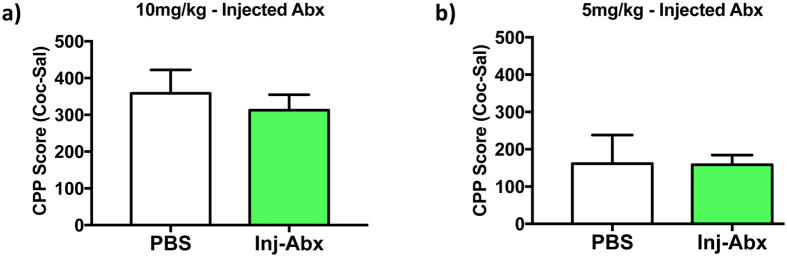
Parenteral administration of antibiotics does not alter behavioral response. For these experiments animals were injected intraperitoneally with phosphate-buffered saline (PBS) or a cocktail of antibiotics (Inj-Abx) for 7 days prior to behavioral analysis. When the conditioned place preference test was performed, there were no group differences when measured at a dose of 10 mg/kg **(a)** or at a dose of 5 mg/kg **(b)**. [n = 5/group].

While our cocktail of antibiotics has been characterized previously as being non- or poorly absorbed from the intestine^4^,^27^,^28^,^29^, we sought to confirm that our behavioral effect was not due to any potential off target effects of the antibiotics. First, to determine if our cocktail of antibiotics was causing the gut to become “leaky” and lead to systemic drug absorption, we measured serum concentrations of vancomycin from animals treated with a prolonged course of oral antibiotics. All 8 of the replicates tested, from a total of 24 antibiotic-treated mice, were fully negative within the limits of this assay (<1.1 μg/ml ± 0). Second, to confirm that the effects of our oral antibiotic treatment were due to local actions within the gut and not due to any unintended systemic effects of the antibiotics, we performed a series of experiments in which mice were injected intraperitoneally with the triple antibiotic cocktail every day – thus bypassing the issue of intestinal absorption. These animals did not show the increased caecal size associated with large-scale knockdown of gut bacteria (data not shown). Since our most robust behavioral effect of oral antibiotics was in the CPP test, we examined effects of parenteral antibiotics in this assay and found no effect of such treatment on cocaine CPP, either at the 10 mg/kg dose (Fig. 4a – two-tailed t test: *p* = 0.57, t = 0.58) or 5 mg/kg dose (Fig. 4b – *p* = 0.97, t = 0.04). These findings support our interpretation that the behavioral effects observed with oral antibiotic treatment are due to depletion of gut microbiota and not due to systemic effects of the antibiotic cocktail.

### Repletion of bacterial fermentation products reverses behavioral effects of antibiotic treatment

**Figure 5:**
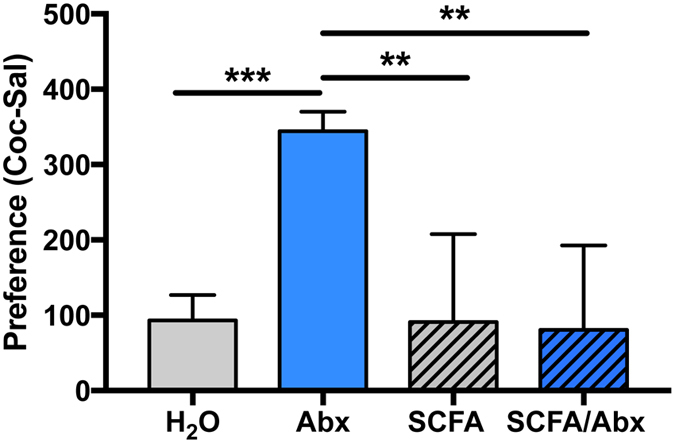
Repletion of bacterial short-chain fatty acids reverses behavioral effect of antibiotics. To determine if the bacterial byproducts were responsible for the changes seen in conditioned place preference (CPP), we measured animals treated with antibiotics (Abx) and/or short-chain fatty acids (SCFA) via the drinking water. Animals were tested for CPP at 5 mg/kg as this was the dose at which a behavioral effect of Abx was seen. As above, Abx treated animals showed a robust preference for cocaine (blue bar). Treatment with SCFA alone did not alter CPP response (grey hatched bar). Animals treated with Abx and SCFA showed the same level of preference as the control animals (blue hatched bar) demonstrating that repletion of bacterial byproducts can reverse the behavioral effects of depleting gut microbiota [n = 5–22/group; **p < 0.01; ***p < 0.001].

Short-chain fatty acids (SCFA) including acetate, propionate and butyrate are major byproducts of bacterial fermentation, and have been shown to play roles in the gut microbiome regulation of microglia, blood brain barrier, immune function and other endpoints^24^,^30^,^31^,^32^. To determine if these metabolites are involved in the behavioral effect of microbiome depletion on cocaine responses, we carried out a series of experiments repleting SCFA in antibiotic-treated animals. We again measured the effect of this manipulation in the 5 mg/kg CPP paradigm, as this was our most robust antibiotic-induced phenotype. As above, animals treated with antibiotics showed a robust preference for 5 mg/kg cocaine, while control animals showed minimal cocaine preference (Fig. 5 grey and blue bars). While SCFA supplementation on its own had no effect on cocaine preference, SCFA supplementation in antibiotic-treated animals resulted in a CPP response equivalent to control mice (Fig. 5 hatched bars; one-way ANOVA F_(3,39)_ = 9.41; *p* < 0.0001 - with Holm-Sidak post-hoc tests). These results are strongly suggestive that these bacterial fermentation products are crucial for the behavioral changes seen in animals with depleted gut microbiota.

### Altered transcriptional response to cocaine in mice with depleted gut microbiota

**Figure 6:**
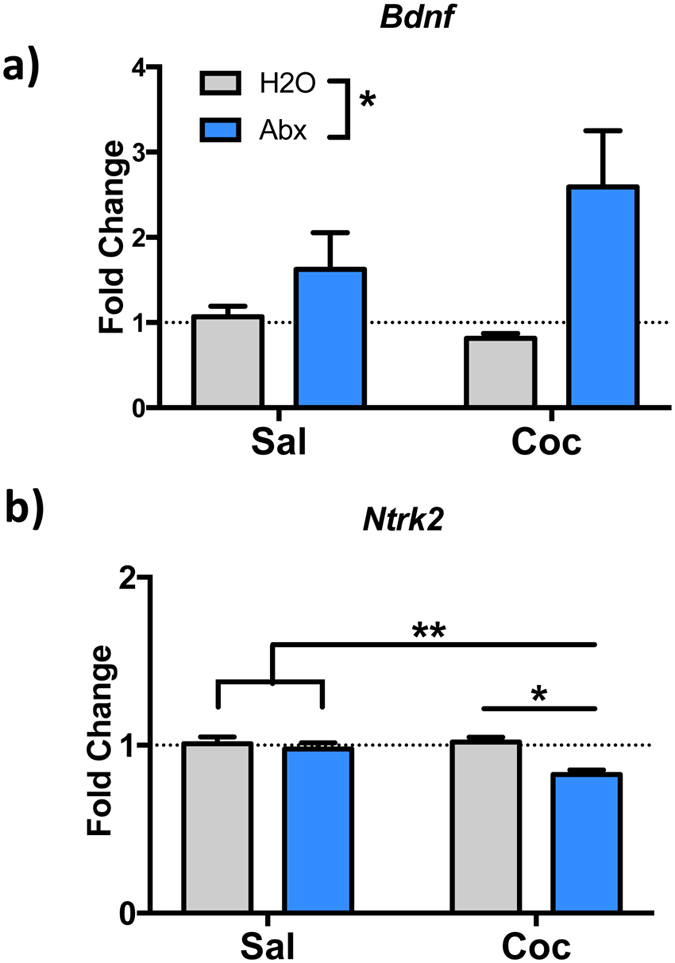
Antibiotic-treated mice exhibit alterations in *Bdnf* and *Ntrk2* transcripts in the NAc. **(a)** In mice treated with antibiotics and cocaine compared to controls levels of Bdnf were significantly elevated in antibiotic-treated (Abx) animals (*p < 0.05; n = 9–11/group); control animals, H_2_O. **(b)** Transcripts encoding the receptor for BDNF were also examined. Levels of *Ntrk2* were significantly decreased specifically in antibiotic and cocaine-treated animals (**p < 0.01; n = 9–11/group).

At this time, the mechanisms underlying microbiota regulation of behavioral responses remain poorly understood. In an attempt to gain some insight into the differences in important brain structures in mice with depleted gut microbiota, we profiled multiple transcripts related to important synaptic plasticity functions involved in cocaine-mediated behaviors. To accomplish this, we examined mice treated with antibiotics and/or 7 days of cocaine (Fig. 1a), and examined transcripts in the nucleus accumbens (NAc)—a critical brain reward region—24 hours after the final injection. As previous studies examining changes in limbic brain structures after alterations in gut bacteria had reported changes in levels BDNF transcript and protein, albeit in other brain regions^4^,^17^,^18^,^19^, we first examined levels of *Bdnf* transcript as well as the transcript encoding the BDNF receptor – Tyrosine receptor kinase B (*Ntrk2*). Levels of *Bdnf* in the NAc were increased by treatment with antibiotics (Fig. 6a - F_(1,37)_ = 7.308; *p* = 0.01), but there was no main effect of cocaine (F_(1,37)_ = 0.6841; *p* = 0.41) or significant treatment interaction (F_(1,37)_ = 2.004; *p* = 0.17). When levels of *Ntrk2* were examined from the same samples there was a significant main effect of antibiotics (Fig. 6b - F_(1,38)_ = 10.31; *p*  = 0.003), cocaine F_(1,38)_ = 4.1; *p* = 0.05), and an antibiotics x cocaine interaction (F_(1,38)_ = 5.377; *p* = 0.03). Interestingly, Holm-Sidak post-hoc analysis shows that this is driven by the Antibiotics+Cocaine group which was significantly different from all other groups in this analysis. These results suggest that depletion of the gut microbiota with oral antibiotics substantially alters BDNF-TrkB signaling in NAc, changes that may well be playing a role in the behavioral changes we see in antibiotic-treated mice.

**Figure 7:**
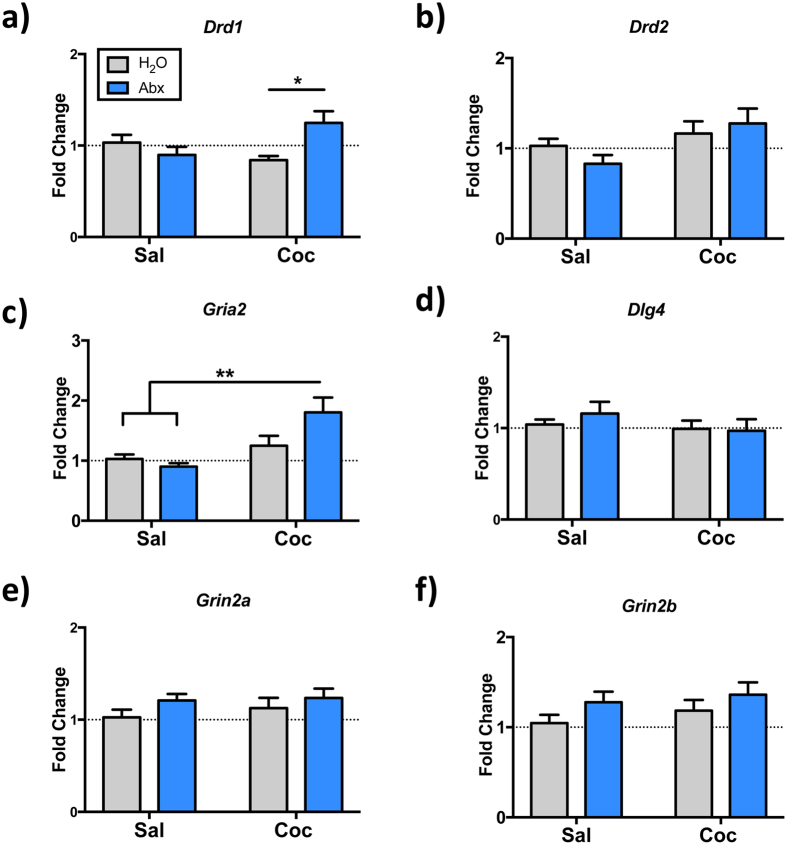
Antibiotic-treated mice have altered expression of dopamine and glutamate-related transcripts in the NAc. Mice with antibiotic-induced reductions in gut bacteria (Abx) demonstrate increased levels of Drd1 (**a**) and Gria2 (**c**) in the NAc following treatment with cocaine; control animals, H_2_O. Levels of Drd2, Dlg4, Grin2a and Grin2b were unaltered. (*p < 0.05; **p < 0.01; n = 9–12/group).

In addition to *Bdnf* and *Ntrk2*, we examined NAc transcript levels of several other synaptic targets known to be important for behavioral response to cocaine. Levels of the transcript for the *Drd1* dopamine receptor did not show a significant main effect of antibiotic or cocaine, but there was a significant cocaine x antibiotic treatment interaction (F_(1,40)_ = 7.74, *p* = 0.008), and post-hoc testing demonstrated a significant difference between the two cocaine-treated groups (Fig. 7a - *p* = 0.04). There were no significant alterations in levels of Drd2 transcript (Fig. 7b). Additionally, levels of the Gria2 transcript, which encodes the GluR2 AMPA receptor, showed a main effect of cocaine (F_(1,42)_ = 11.96; *p* = 0.001) and a cocaine x antibiotic interaction F_(1,42)_ = 4.43; *p* = 0.04) with post-hoc testing demonstrating significant difference between the antibiotics + cocaine treatment group and both saline treated groups (Fig. 7c). There were no significant differences in levels of *Dlg4* (which encodes postsynaptic density protein 95 or PSD-95) or the NMDA receptor transcripts *Grin2a* and *Grin2b* (Fig. 7d–f).

## Discussion

In this study we find that depletion of the gut microbiota results in a marked change in behavioral responses to cocaine in multiple paradigms. This suggests that interactions along the gut-brain access are important for development of cocaine reward associations as well as sensitized behavioral responses to cocaine. While the mechanistic underpinnings that are responsible for these behavioral changes are not fully clear, we found that depletion of gut bacteria resulted in altered transcriptional profiling in the NAc, an important area for the development of both place preference and locomotor sensitization. Together these data provide important insights into the addiction process as well as provides novel targets for potential treatment interventions to improve treatment outcomes in drug addicted individuals.

We see that depletion of the gut microbiota caused enhanced sensitivity to the rewarding and sensitizing properties of cocaine (Fig. 3). This represents the first report that alterations to the gut microbiome can affect behavioral response to psychostimulant drugs. Previous studies on the effect of the gut microbiome on behavior have focused primarily on models of anxiety and depression^5^,^6^,^22^,^33^. While there is clearly overlap between mood/anxiety and substance use disorders^14^,^15^, characterization of how gut microbes can directly affect drug-related behavior represents an important step forward. A series of studies has shown that a subset of patients with alcoholism have increased gut dysbiosis and that this is associated with increased depressive symptoms and cravings to drink^34^,^35^. While this does provide compelling evidence of a correlation between gut dysbiosis and alcohol action, studies providing a direct examination of the two have yet to be published. The changes we see in the altered sensitivity to rewarding and sensitizing effects of cocaine are important, as they suggest that alterations in gut bacteria can change cocaine-related behaviors. There is already one study in humans demonstrating that subjects with cocaine use disorder have alterations in their gut microbiota^13^. Together with our findings, this suggests the possibility of the gut microbiome as a druggable target for preventing or treating psychostimulant addiction.

While this is the first study to directly examine the role of the gut microbiome in cocaine-mediated behavioral plasticity, it is not the first to use antibiotics to alter behavioral responses to cocaine. Multiple previous studies demonstrated that systemic injections of cephalosporin antibiotics decrease reinstatement of cocaine self-administration^36^,^37^,^38^, possibly through upregulation of the GLT-1 glutamate transporter at the synapse^39^. However, none of these studies examined changes in levels of gut bacteria, so the possible involvement of the gut microbiome in cephalosporin actions is unknown. In our study we saw behavioral effects of antibiotics only when they were given orally (Fig. 3), and thus were directly applied to the gut microbiota. Systemic injections of the antibiotics used in our study did not lead to any behavioral phenotype (Fig. 4), suggesting that it was their ability to reduce gut bacteria that lead to their behavioral effects.

The goal of these experiments was to assess the effect of a substantially reduced gut microbiota on mouse behavior with the fewest possible confounding effects. While a number of studies have utilized daily gavage feeding of antibiotics to reduce gut microbiota^1^,^23^, a method that provides finer control over the daily dose each animal receives, we were concerned that the stress of daily forced feedings would cloud our ability to interpret the behavioral results^40^. Gavage feeding is a stressor intense enough that it was used to induce a depressive-like phenotype in a recent publication^8^. Several other studies have relied on germ-free mice that are devoid of commensal bacteria from birth^41^. While these animals do provide the cleanest model system for modeling the effect of gut bacteria on host physiology, there are myriad developmental effects of complete lack of commensal microorganisms. At this time there are known developmental effects on the immune system^42^,^43^, gastrointestinal system^2^, as well as multiple effects in the CNS including altered patterns of myelination^44^, function of microglia^24^, and permeability of the blood brain barrier^32^, among others.

Fortunately, we were able to administer these non-absorbed antibiotics via the drinking water in a manner that allowed the animals to maintain normal fluid intake and body weight, but significantly depleted the intestinal microbiota (Fig. 1). Importantly, these effects are independent of potential effects of antibiotic treatment on cocaine metabolism. Given that cocaine is primarily metabolized by the liver, it was important for our purposes to know that the alterations of gut microbiota did not affect the metabolism of cocaine – which could have affected the effective dose of cocaine each animal received. When we assayed for serum benzoylecygonine, the primary cocaine metabolite that is present in the blood, in a time course after high or low dose cocaine doses we saw no differences in control and antibiotic-treated animals (Fig. 2a,b) – suggesting that our strategy to reduce gut microbiota did not affect cocaine metabolism through either direct or indirect mechanisms. Similarly, the dramatic reduction in gut bacteria could have potentially resulted in alterations in the HPA axis which could have had confounding effects on our behavioral models. However, when serum levels of corticosterone were measured after a prolonged course of antibiotics there were no significant effects of antibiotic treatment on levels of this stress hormone (Fig. 2c). Based on this, it seems that these studies provided for a model in normally developed mice with a minimum level of added stress.

While the mechanism of how gut microbiota affect behavior remains to be elucidated, we show here that repletion of the bacterial fermentation products the SCFAs reverses the antibiotic-induced behavioral phenotype (Fig. 5). Interestingly, several recent studies have shown that SCFAs are a key component in the microbiota regulation of brain function^24^,^32^. While the mechanism of action of the SCFAs on cellular and behavioral function is not fully known there are several possibilities. Of the three main SCFAs produced in the gut (butyrate, propionate, acetate), two are known to be histone deacetylase inhibitors (HDACs)^30^. Changes in epigenetic histone acetylation are well characterized as playing a role in the behavioral responses to cocaine^45^,^46^. Studies have shown that chronic administration of HDAC inhibitors reduces behavioral responding for cocaine^45^,^47^,^48^. An important goal for future research will be to study the possibility that knockdown of the gut microbiota reduces production of SCFAs, which act as HDAC inhibitors leading to increased behavioral sensitivity to cocaine^49^.

To examine molecular changes associated with the changed behavioral sensitivity to cocaine, we assessed the transcriptional profile of a number of gene targets in the nucleus accumbens. In our antibiotic-treated animals given cocaine, we see alterations in the transcripts encoding both BDNF and its TrkB receptor (Fig. 6). BDNF signaling in the NAc has been repeatedly reported to alter behavioral responses to cocaine^50^,^51^. Interestingly, numerous other studies examining the behavioral effects caused by alterations in the gut microbiome have also found changes in levels of BDNF in multiple brain structures^4^,^17^,^18^,^27^. While those studies were examining other brain regions and behavioral paradigms, the consistent finding that altered gut microbiota results in altered BDNF signaling lends credence to the idea that this is one of the ways in which changes in gut microbiota may be affecting behavior.

In addition to changes in BDNF-related gene function, we also found alterations of several other important transcripts in the NAc (Fig. 7). We see elevations in *Gria2*, the transcript encoding the GluR2 subunit of the AMPA glutamate receptor, in animals treated with both cocaine and antibiotics. Previous studies have shown that cocaine induction of GluR2 in this brain region is downstream of the transcription factor ∆FosB, and that overexpression of GluR2 in the NAc increased behavioral responses to low doses of cocaine, similar to effects seen in our animals^52^. Additionally, increased surface expression of AMPA glutamate receptor subunits is seen in animals expressing behavioral sensitization after prolonged periods of withdrawal^53^. In addition to elevations in the *Gria2* transcript, animals treated with antibiotics and cocaine showed a significant elevation in *Drd1,* the transcript encoding the D1 dopamine receptor. Signaling via D1 dopamine receptors is known to be crucial for the reward mechanisms underlying cocaine abuse^54^,^55^. Additionally, activity of the NAc medium-spiny neurons expressing these receptors is crucial for the formation of both locomotor sensitization and conditioned place preference^56^,^57^. While the current study examined only transcriptional changes in these patterns, they suggest that both glutamatergic and dopaminergic transmission systems may be playing a role in the behavioral alterations seen in microbiome-depleted animals.

Although further work is needed to understand the underlying mechanisms, results of the present study define clear paths forward for future research. In particular, it will be important to define how alterations in gut microbiota interact with cocaine treatment to modify the full transcriptome and protein expression in NAc and other important limbic reward structures. Additionally, future studies will include examination of the effect of altered gut microbiota on cocaine self-administration and reinstatement. Studies on probiotics, prebiotics, and bacterial metabolites can be utilized to determine how manipulations of the microbiome may reduce cocaine taking and seeking behaviors. While much remains to be examined, there is clearly tremendous potential for future translational studies in this emerging area of microbiota-gut-brain axis communication.

## Methods

### Animals and Drug Treatments

Male C57BL/6J mice aged 7 weeks were purchased from Jackson Laboratories and were group housed under specific-pathogen free conditions on a 12-hr light/dark cycle (lights on 7:00 am-7:00 pm). All animals were allowed to acclimate to the facility for one week prior to the start of any treatments. Antibiotics were administered via the drinking water in an adaptation of a previously published protocol that has been shown to substantially deplete the gut microbiota^4^. Antibiotic doses were: Bacitracin 0.5 mg/ml, Neomycin 2 mg/ml, Vancomycin 0.2 mg/ml and Pimaricin 1.2 μg/ml. Concentrations were adjusted from the cited study as our animals did not drink the higher concentrations; vancomycin was added to increase coverage of gram positive organisms. Doses based on mean daily intake were calculated to be at or above what would normally be given to treat gastrointestinal infections. These agents were specifically selected because they are well characterized as being non-absorbed from the intestine^4^,^27^,^28^,^29^. This allows for maximal debulking of gut bacteria and minimal off-target effects due to systemic absorption. The antibiotic mixture was changed every two days and animals were weighed during the first week of experimentation to ensure body weight was maintained. Animals remained on antibiotic treatments throughout all cocaine treatments and behavioral paradigms. To measure any behavioral effects of parenterally-administered antibiotics we injected animals with 1.67 mg/kg vancomycin, 20 mg/kg neomycin, and 293 U/kg bacitracin in PBS every day for seven days prior to behavioral testing. During days of behavioral testing injections were performed >2 hours prior to the start of testing to avoid any association of the injection with the chamber. For experiments with short-chain fatty acids (SCFA) animals were given three days of antibiotic or control treatment, followed by addition of the three principle SCFAs produced by bacteria at concentrations similar to those found endogenously in the gut (67.5 mM acetate, 40 mM butyrate, 25.9 mM propionate) as has been reported in multiple previous studies examining effects of SCFA in mice^31^,^32^. All animal protocols were approved by the Institutional Animal Care and Use Committee (IACUC) at Mount Sinai. All animal experiments were performed in accordance with the National Insitutes of Health Guide for Care and Use of Laboratory animals as well as with policies of the IACUC of the Icahn School of Medicine at Mount Sinai.

### Benzoylecygonine Elisa

To determine if treatment with antibiotics altered metabolism of cocaine, levels of benzoylecygonine, the primary metabolite of cocaine, were measured in serum of acutely treated animals. For these experiments Control or Antibiotic-treated animals were given a single injection of 20 or 5 mg/kg cocaine. Animals were killed by rapid decapitation at 30, 60, or 120 minutes after the injection of cocaine. Trunk blood was allowed to sit at room temperature for at least one hour to allow for full clotting. Samples were then spun at 1,200 × *g* for 15 minutes and the supernatant was saved as serum. Serum concentration of benzoylecygonine was measured using a direct benzoylecygonine ELISA (Sigma-Aldrich) according to manufacturers instructions.

### Corticosterone ELISA

To measure the effect of prolonged antibiotic treatment on levels of serum corticosterone animals were treated with antibiotics via their drinking water for two weeks as described above. Animals were then analyzed in the afternoon to correlate both with peak levels of the hormone and with the time of day that behavioral endpoints were being measured. Serum was processed as above, and corticosterone concentrations were measured using a commercially available ELISA kit (Abcam). Serum samples were diluted 1:100 per manufacturer instructions.

### Serum vancomycin measurements

To determine if prolonged antibiotic treatment caused the absorption of antibiotics that are not absorbed in normal animals, we treated animals for two weeks with the triple antibiotic cocktail via their drinking water as described above. Serum was collected as above. Serum samples from 24 mice were collected to form 8 replicates which were sent for testing in the Mount Sinai Hospital clinical laboratory. There they were assayed according to their standard vancomycin immunodetection assay according to the same protocols used to monitor therapeutic vancomycin concentrations in patients. The assay has a lower limit of detection of 1.1 μg/ml.

### Locomotor Activity Analysis

Locomotor activity after repeated cocaine was measured similar to previously published reports^58^. Activity was monitored in rat sized cages with no bedding set up in a locomotor apparatus and evaluated on extent of ambulations in the x and y planes. On the first three days of each experiment the animals were given an intraperitoneal (i.p.) injection of normal saline and locomotor activity was monitored for 45 minutes. For each of the next five days animals were injected with i.p. cocaine (5 or 10 mg/kg) and their activity monitored for 45 minutes.

### Conditioned Place Preference

An unbiased conditioned place preference (CPP) assay was carried out as described previously^49^,^56^,^59^. Mice were evaluated for cocaine place preference using 3 chambered CPP Med Associates boxes and software. The two end chambers were made to have distinct visual (gray vs. striped walls) and tactile (small grid vs. large grid flooring) cues to allow differentiation. On the pre-test day animals were allowed to freely explore all three chambers for 20 minutes and those that showed a significant preference for one of the two chambers were excluded from further analysis (<10% of animals tested). Groups were then balanced and adjusted to balance out any pre-existing chamber bias. Place preference conditioning was carried out by pairing an injection of saline with one chamber in the morning, and a second injection of cocaine (5 or 10 mg/kg) with the other chamber in the afternoon for two consecutive days. CPP testing was carried out on the fourth day when each animal was again allowed to explore all chambers freely. Place preference score was taken as time on the cocaine paired side – time on the saline paired side.

### RNA Isolation and qPCR from brain tissue

For biochemical experiments animals were treated with high dose cocaine (20 mg/kg/day i.p.) and killed 24 hours after the final dose of cocaine. Nucleus accumbens tissue was rapidly dissected and frozen on dry ice. RNA isolation, qPCR and analysis were done as previously described^60^. Briefly, RNA was extracted by homogenizing tissue in Qiazol reagent (Qiagen) and purified using RNeasy micro kits from Qiagen according to manufacturer protocols. RNA concentration and quality was assessed using a NanoDrop spectrophotometer (Thermo). Reverse transcription was performed using iScript (BioRad). qPCR using SYBER green master mix (Quanta) was carried out using an Applied Biosystems 7900HT cycler with the following parameters: 2 min at 95 °C; 40 cycles of 95 °C for 15 s, 59 °C for 30 s, 72 °C for 33 s; and graded heating to 95 °C to generate dissociation curves to confirm amplification of a single PCR product. Data were analyzed by comparing C(t) values of control and antibiotic treated mice using the ∆∆C(t) method^61^. Primer pairs used for analysis are available in Table 1.

### qPCR for intestinal bacteria

Samples from the cecum were taken at the time of sacrifice and bacterial DNA was extracted with the powersoil DNA isolation kit (MoBio) according to manufacturers instructions. SYBR green-based qPCR was performed using universal eubacterial primers that have been previously published and characterized^24^,^62^. PCR cycling was carried out in the same manner as in qPCR from brain tissue. Fold change in total bacterial load was calculated by normalizing to DNA content as described previously^24^.

### Statistical Analysis

Statistical analysis and figure preparation were performed using GraphPad Prism v7. Pairwise comparisons were performed using unpaired two-tailed t-tests, two-by-two comparisons were performed using two-way ANOVA with Holm-Sidak post-hoc tests.

## Additional Information

**How to cite this article**: Kiraly, D. D. *et al*. Alterations of the Host Microbiome Affect Behavioral Responses to Cocaine. *Sci. Rep.* **6**, 35455; doi: 10.1038/srep35455 (2016).

## Figures and Tables

**Table 1:** Primers used for quantitative PCR reactions.

Target	Forward	Reverse
*Bdnf*	CCATAAAGGACGCGGACTTGTACA	AGACATGTTTGCGGCATCCAG
*Ntrk2*	TTGTGTGGCAGAAAACCTTG	ACAGTGAATGGAATGCACCA
*Drd1*	GCTGGCTTTTGGCCCTTTGGGTC	GCTGGAGATAGCCCAATACCTGTCC
*Drd2*	CCATCTCTTGCCCACTGCTCTTTGG	GGTGACGATGAAGGGCACGTAGAAC
*Gria2*	TGCGACACCATGAAAGTGGG	AAAGACACATCAGGGTAGGTGG
*Dlg4*	CACCCTAGAAGCCCCAGGAT	AAGATGGATGGGTCGTCACC
*Grin2a*	TCCGCCTTTCCGATTTGGG	GCGTCCAACTTCCCAGTTTTC
*Grin2b*	CTGAGACTGAAGAACAGGAAGATGACCATC	CGGGACTGTATTCCGCATGCAGG
*Gapdh*	TTGTCAGCAATGCATCCTGCACCACC	CTGAGTGGCAGTGATGGCATGGAC
Univ. Eubac.	ACTCCTACGGGAGGCAGCAGT	ATTACCGCGGCTGCTGGC
